# Cognitive Based Authentication Protocol for Distributed Data and Web Technologies

**DOI:** 10.3390/s21217265

**Published:** 2021-10-31

**Authors:** Natalia Krzyworzeka, Lidia Ogiela, Marek R. Ogiela

**Affiliations:** 1 AGH University of Science and Technology, 30 Mickiewicza Ave., PL-30-059 Kraków, Poland; nkrzyworzeka@gmail.com; 2 Pedagogical University of Krakow, Podchorążych 2 Street, PL-30-084 Kraków, Poland; lidia.ogiela@gmail.com

**Keywords:** personal CAPTCHA, visual CAPTCHA, login scheme, password reminder, cognitive processes

## Abstract

The objective of the verification process, besides guaranteeing security, is also to be effective and robust. This means that the login should take as little time as possible, and each time allow for a successful authentication of the authorised account. In recent years, however, online users have been experiencing more and more issues with recalling their own passwords on the spot. According to research done in 2017 by LastPass on its employees, the number of personal accounts assigned to one business user currently exceeds 191 profiles and keeps growing. Remembering these many passwords, especially to applications which are not used every week, seems to be impossible without storing them either on paper, in a password manager, or saved in a file somewhere on a PC. In this article a new verification model using a Google Street View image as well as the user’s personal experience and knowledge will be presented. The purpose of this scheme is to assure secure verification by creating longer passwords as well as delivering a ‘password reminder’ already embedded into the login scheme.

## 1. Introduction

According to recent research from 2020, over 80% of data breaches are caused by stolen credentials or brute force attacks [1,2]. Using weak passwords, such as ‘qwerty’, or reusing the same password across multiple websites is considered to be one of the major factors that weaken account security. 

One of the possible solutions to this problem is to promote by application providers the use of longer and more complicated passwords. However, in case of non-dictionary passwords with large entropy, it may become impossible for users to remember them, especially when we consider that around 191 profiles need such credentials. 

Password managers could help users store their passwords, although these types of applications are usually not free of charge and, in case of multiple devices, they need to be installed on each of them.

The goal of this paper was to propose a login scheme that could play a role of a password reminder, while helping users in generating passwords which are longer and more secure than average. Longer passwords guarantee a higher level of security, which results directly from t information theory, as well as the theory of complexity. Longer passwords may have a higher entropy (bits of important information), which makes cryptanalytic attacks significantly more difficult, and considerably increasing the time of finding them by using the brute-force method. The solution we present will significantly increase the entropy of passwords that are generated with the use of the proposed algorithms. 

The method relies on a person knowing a specific street or area that could be observed e.g., on a Google Street View^®^ user, when presented with an image of this street, is asked to provide a password, that should be a combination of a street name or presented building, the name of the city in which the depicted street is located or a custom word and a number. A combination of all three of these elements should result in a longer than typical password.

This specific part of knowledge, recognising a location special to us that will not be obvious to others, can help distinguish the user who created this particular login scheme from any other person. Choosing an image with a known street for the login scheme should be based on our own personal preferences and memories. 

Visual memory has been proven to be stronger than audio memory [3] and when associated with emotions, positive or negative, it can enhance memory for those stimuli [4]. Therefore, remembering the name of the place, e.g., one in which we had our best vacations, and about which we are having strong and good memories, should let us more easily recall the name of the place which we ourselves also picked.

In the US alone, there are estimated to be around 240 million streets, and given that its population is around 330 million people, we could say that each street could be picked not more than two times by someone in the country. The strength of this method relies on the user realizing the consequences of picking a well-known location and on him/her picking the image from Google Street View^®^ best suited for this task. 

This Google^®^ Database was chosen in this article because of its free access, size, quality of images and availability. Snapshots from Google Street View^®^ however, cannot be directly shown to users without pre-processing, since names of the streets are already embedded intothe Street View. That would be an obvious hint for the password. This article solves this problem by proposing an algorithm that would quickly cover the street name.

In order to create an optimal and safe solution, this paper presents CAPTCHA (Completely Automated Public Turing test to tell Computers and Human Apart) algorithms based on a password reminder. Passwords reminders will be based on intelligent associations with a known place and in some way related to a given person. Therefore, cognitive methods will be used to associate a given password with its specific location, known only to the user [5,6,7,8].

The structure of this paper is the following: Section 2 presents a state of the art and related papers. Section 3 presents the main idea of an intelligent personal CAPTCHA based on specialist knowledge. Section 4 presents a cognitive CAPTCHA Password Reminder based on individual associations and unique personal experiences. 

Section 5 presents the safety of proposed solution, and Section 6 describes some conclusions and future work.

## 2. Related Works

Login credentials are almost always a combination of a unique username and a password. A password is nothing else but some sort of information that only one user would know or be in possession of, e.g., a work badge, a fingerprint, or a text-based or voice-based sentence. Almost all computer applications now rely on text-based passwords due to the simplicity of their implementation. This forces users to remember lengthy, usually semi-random sentences with numbers, that should be hard to guess by anyone. Taking into consideration that human memory has limited capacity and can be in some cases erased or modified, it is becoming harder and harder to remember and provide on-demand correct passwords for each of our growing number of personal accounts.

Password-guessing and phishing are two of the most successful types of attacks. Protection against password guessing is suggested in a work [9] that analyzes the strength, and more precisely the entropy, of a secure password. Their suggestion was to create around 21-character long phrases with non-alphanumerical characters and capital letters. Words used in that password also have to be used in a way that a dictionary of more than 50,000 words would not contain them [9]. Creating this complex combination of symbols should not be hard to remember if it was only for one application, but what to do in case 100 or more accounts require this level of security?

Researches have proven that users re-use their passwords in various ways [10]. Their original “password base” is usually lengthened or modified, depending on the level of criticality of data that it is protecting. The same studies have also shown that in case a user’s lower-level password is leaked, it can be successfully exploited by an attacker to crack some of the user’s higher-level passwords [10]. Some researchers provide guides for users that should help them recall their passwords more efficiently, e.g., mnemonic password or password chunking [11]. This however requires special dedication from an interested party, and may not be considered a necessity by a regular user.

Problems with remembering passwords have been a subject of study for many years now. There were many successful attempts to introduce non-biometric or password-less methods, such as visual cryptography [9]. The downside of these solutions is often them being device-dependent, since private keys are not stored everywhere. Human memory still seems to be the most secure and confident space for password storage.

## 3. Personal CAPTCHA

Personal log-in schemes, presented in this article, are a step further from personal CAPTCHA. Personal CAPTCHAS are part of an authentication process and are tailored for certain groups of people or for a specific user. Their purpose is to raise the difficulty of the CAPTCHA scheme for everyone except their target group. 

Due to ever growing advances in technology, especially machine learning, it has become harder for CAPTCHA engineers to design a scheme that is easy to solve by every human and impossible to pass for automated programs. Personal CAPTCHAs allow for increasing scheme difficulty for non-target users or bots, but do not appear to be any more problematic for their target group. An example of a personal CAPTCHA could be a scheme that contains discipline-oriented questions that would allow for easier verification for a group of students or professors of a university [12].

A personanllogin-scheme must consist of a task that is solved only by one user. Designing this kind of task seems to be impossible, unless we take the user’s personal preferences, knowledge and visual memory into account [13,14,15]. Creating such a scheme for each site member can also be unrealistic, but not when users themselves are the ones doing all the work: picking the scheme image, making their own visual word associations and finally creating a password.

The scheme presented in this article is verifying only one of many possible pieces of knowledge that could be used in this kind of personal login-scheme. Personal CAPTCHAs and personal login-schemes not only make it easier for users to pass the verification process, but also to increase their security.

## 4. Proposed Cognitive CAPTCHA Password Reminder

When creating an application account, a user is asked to provide a snapshot from Google Street View^®^ with a location of his/her choice [16]. There also could be an already programmed website feature that would allow users to choose the street from the map on the site itself. 

For implementation, website providers can pick any of the free Google Maps plug-ins available online [17], which is why this paper will focus only on the login scheme with an already obtained street image.

The essential thing to remember is that in order for the scheme to have the desired level of security, users have to be advised to pick a not well-known street or building

Ideally, the depicted place should be far away from one’s house or workplace, so no one from their areas is able to recognize it. Picking subjectively characteristic or sentimental areas is also a good choice because it would enable users to recognize it even after a couple of years.

It is advised to pick a not well-known street or building, ideally far away from our house or workplace, one which we shall have no problem recognising even after a couple of years. It could also be a good idea to select only a small fragment of a house or tree that is in some way very distinctive to us. By doing this we broaden the possibilities of the search area against possible brute force attacks. It may also be harder for a random person to guess the location, since there is less information provided in the image., Users would be encouraged to select streets according some past recollection and good memories, which are very specific and unique for particular user. Good memories can be dependent also on lifestyles and habits.

**Figure 1:**
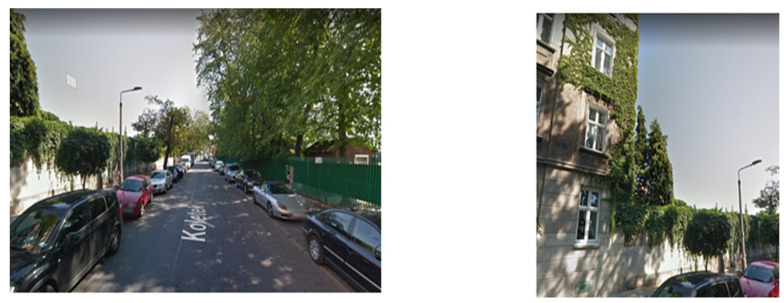
Image from Google Street View of a street and building from the different views.

Figure 1 shows an example of a street view and a building view from the same location. For some users taking a snapshot of a building will be more helpful, since it has more distinct features, and assuming that we have seen it many times in our life, it should be easier to recall (Figure 1).

After the user has selected a file, the next step for the algorithm is to remove the street name, visible usually down in the middle and embedded into the road ahead, as well as other Google markings and tags. The implementation was done in Mathlab version 2010a on Windows10. The proposed program first crops the photo to a standard size, since all markings (like directions and copyright information) are always the same size and position. Next, an algorithm detects the letters, using threshold-based methods, by taking advantage of the fact that the street name is showing as all-white letters embedded into the road. Then with the use of a dilatation-based algorithm, shown also in [4], the writing would be distorted. In this process each pixel from the distorted area is assigned a random colour in order to further cover up the writing. The program could also have a feature that would calculate the range of pixels depicting a road or asphalt, making the removal less evident.

There is also an option to obtain graphics without the street name embedded, just by moving Google Street View off the road to the left or right view and then taking a snapshot.

**Figure 2:**
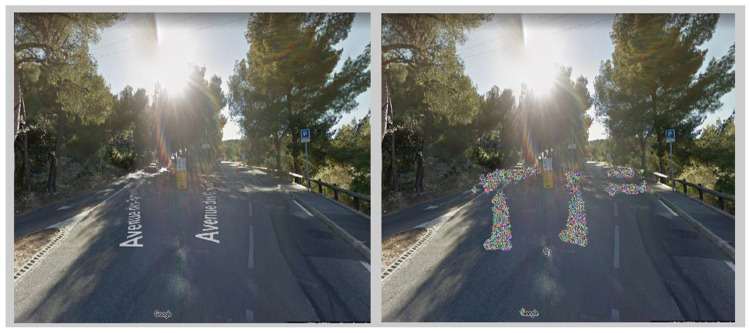
Google Street View before (**left**) and after (**right**) removing the embedded street name.

Figure 2 presents an image before and after the removing of street name by the described algorithm.

**Figure 3:**
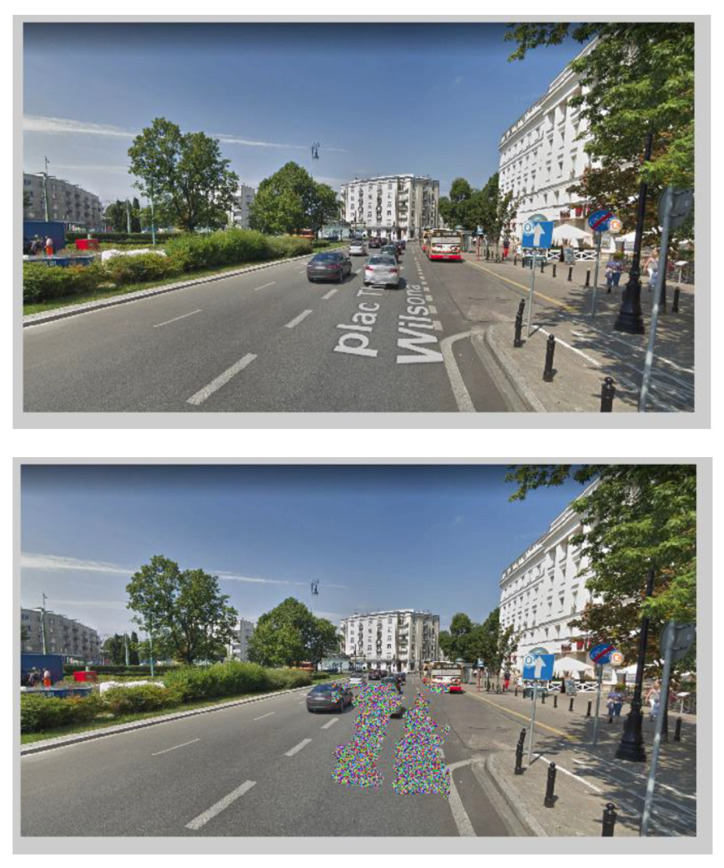
Example of an image obtained by providing GPS coordinates.

The presented scheme could also be programmed in a way that the user would only provide the street name and city or GPS (Global Positioning System) coordinates. If he/she is familiar with the whole area, there would be no problem with the website presenting a different view of the street during each login, randomly obtained from Google. The street view from Figure 3 shows an image obtained by only providing GPS coordinates, and its processed version.

After removing the embedded name from the image, the user is asked to create a password that has to have at least 9 characters, which is a standard password requirement. The password must consist of a street/object name, visible on the image, a number (e.g., building number or area code) and optionally another word. The third word that should be added could be a city name or phrase that the user will have no problem recalling. 

**Figure 4:**
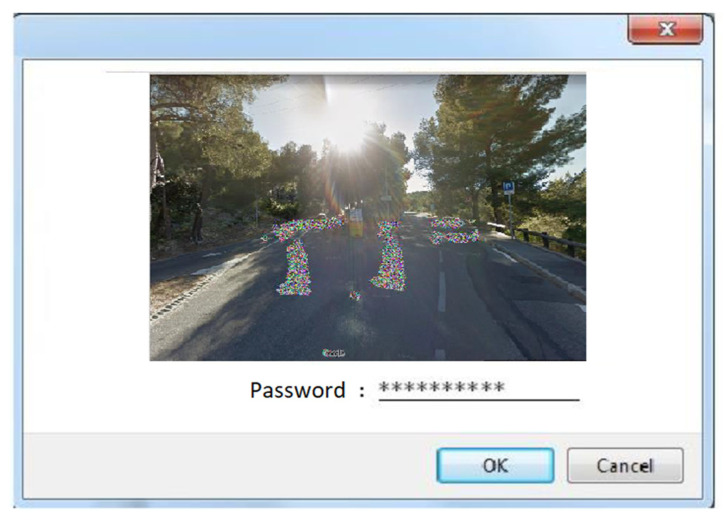
Login scheme using Google Street View image.

The next time, after providing the login information, the user will be shown his/her unique password reminder scheme and will be asked to provide a password. An example of a login scheme is presented in Figure 4.

## 5. Discussion—Safety of the Proposed CAPTCHA Password Reminder

The safety of the developed algorithm is based primarily on individual, personal associations of a given street/location. Personal associations of a specific location are individual and characteristic for a given person and are unique. Cognitive aspects of the description of a specific place allow for its unambiguous identification, and by linking it with specific impressions/experiences, they allow them to be retained in the memory for a long time, which makes them quickly recalled.

Assuming that the shown street is not at all known to a random person, the view of the street itself should not give any hints about the password. Since street names are often very long, they can contain several words and are therefore not in the dictionary, therefore this should help protect users against brute force attacks on their accounts [18,19]. Most websites are already blocking specific IP addresses from logging in after several unsuccessful password attempts, which will protect users from attacks of this kind. Knowing this, bots are often providing most ‘common passwords’ for a list of users, in hope they would match with one account. When using the described method, it should be almost impossible to guess correctly the street name, number and another key word for a particular account. 

It is also not likely that hackers would try an exhaustive search of the particular Google Street View image, e.g., of the city in which the user is from. That is because Google will most likely block any IP address that sends too many requests in a short time from a specific IP address. Such action will most likely be seen as ‘attack’ and ultimately it will not be successful. Google Maps contains around 21 million gigabytes, or around 20,500 terabytes of data. This amount of data will be almost impossible to analyse in search of a direct correlation with the user’s image. This is also due to the fact that our street name has been distorted and the image was cropped, which would also involve recalling and object recognition algorithms.

The use of proper names of streets, cities, and regions in key generation and user authentication can be realised in a completely unambiguous way even if different alphabets and letters are used. The unification of names can be done easily by image processing and classification procedures of these names. The user, while analysing them, may freely recognise various forms of used names, as well as their abbreviations, especially when it is the user who chooses an image presenting the preferred fragment of the street. 

In the described procedure of password generation using images of selected streets or places, an attack in the form of user impersonation by relatives may occur. In order to make such an attack more difficult, when choosing the preferred images, one should avoid patterns in the closest environment, which may evoke similar associations in close relatives. The use of other patterns, recognisable only by an authorised user, guarantees that the generated password will be safe.

The password reminder system presented here is a solution oriented to password generation or restoration with relation to the personal memory and cognitive skills of an individual user. It is quite convenient and can be applied in distributed technologies as well as in web browsers. In this case a module which can serve as reminder should be added to the user’s computer, which could also, for instance, store user streets preferences chosen during password generation. This solution can be implemented in the same application as the newest WebAuthn technologies, which are mostly oriented on user authentication based on public key cryptography.

## 6. Conclusions

The login scheme presented in this paper, which also plays the role of a password reminder, could be featured on any website. Passwords created with the use of this method should not only have a greater entropy than standard passwords, but also should be much easier to remember. Associating an image with a person’s personal experiences and memories gives a unique bond to a created password that cannot be imitated or reproduced by anyone else. 

There are currently no plans for creating a tool which could assist users by checking a password’s complexity and strength, e.g., typing keywords from user’s password into the Google search and checking if it matches graphics similar to the image chosen for scheme. This is because it could violate the user’s privacy and lengthen the registration process. Users themselves should be sufficiently aware of possible consequences of creating weak passwords.

A user’s awareness of the importance of non-dictionary passwords and their length grows bigger each year, and we can safely assume that with the help of additional images, each person should be able to create more complex passwords.

The greatest downside of the presented method is the additional step during account creation, which the user has to take by providing a street image. Time spent on picking up such a graphic should not take long and possibly will be paid back in the future, since users should more rarely use the option requesting a password hint with this scheme.

Another downside of the presented method is increasing the website’s database size. Application providers must reserve memory for all of the profile’s images. Quality of street photos does not have to be high for users to recognise them, but also should not be compressed too much to avoid possible ‘bad guesses’ from a user in case he or she forgets which street was set for which account.

This intelligent CAPTCHA model might be simply applied when password regeneration is needed, substituting traditional important questions chosen by the user at the time of registration.

Compared to other solutions widely used in web browsers and key management procedures, our approach is different as not only based on traditional cryptographic solutions. In particular, it can be used as a password manager, which is oriented to a particular user, and connected with his/her perception skills and experiences (life, professional etc.). It is also connected with the cognitive features and memory functions of a particular user. On the other hand, generated passwords are strong enough thanks to the connections of input sequences with image parameters selected by users. Beside password generation it can also be used for password restoration with relation to the personal cognitive features. 

This password reminder has been implemented and tested on several PC machines running under the Windows 10 operating system. It has been tested mainly as a new scientific proposition, and in the near future after standardization may be developed in real protocols and services. This method should be evaluated by a group of independent end-users that that would evaluate its difficulty, user-friendliness and whether it would help encourage them in creating more difficult website passwords. This is a first step of continued future work by these authors. This approach will be also extended towards automatic selection of visual CAPTCHA elements which can improve the efficiency of authentication protocol [20], and application in IoT areas [21,22]. The automatic generation of visual patterns will be implemented especially with regard to the application of cognitive vision systems, allowing for the collection and generation of thematic sequences of visual patterns connected with selected areas of interest. Similar solutions were applied in the generation of cognitive CAPTCHA [5].

## Data Availability

Data sharing not applicable.

## Abbreviations

The following abbreviations are used in this manuscript:

CAPTCHA	Completely Automated Public Turing test to tell Computers and Human Apart
GPS	Global Positioning System
